# The context signals of mitochondrial miRNAs (mitomiRs) of mammals

**DOI:** 10.18699/VJGB-22-99

**Published:** 2022-12

**Authors:** O.V. Vishnevsky, P.S. Vorozheykin, I.I. Titov

**Affiliations:** Institute of Cytology and Genetics of the Siberian Branch of the Russian Academy of Sciences, Novosibirsk, Russia; Novosibirsk State University, Novosibirsk, Russia; Institute of Cytology and Genetics of the Siberian Branch of the Russian Academy of Sciences, Novosibirsk, Russia Novosibirsk State University, Novosibirsk, Russia Kurchatov Genomic Center of ICG SB RAS, Novosibirsk, Russia

**Keywords:** miRNA, pre-miRNA, mitomiR, mitochondrion, микроРНК, пре-миРНК, митомир, митохондрия

## Abstract

MicroRNAs (miRNAs) are small non-coding RNAs that regulate gene expression at the post-transcriptional level in the cytoplasm and play an important role in a wide range of biological processes. Recent studies have found that the miRNA sequences are presented not only in the cytoplasm, but also in the mitochondria. These miRNAs (the so-called mitomiRs) may be the sequences of nuclear or mitochondrial origin; some of them are involved in regulation of the mitochondrial gene functions, while the role of others is still unknown. The identification of nucleotide signals, which are unique to mitomiRs, may help to determine this role. We formed a dataset that combined the experimentally discovered mitomiRs in human, rat and mouse. To isolate signals that may be responsible for the mitomiRs’ functions or for their translocation from or into mitochondria a context analysis was carried out for the sequences. For three species in the group mitomiRs/non-mitomiRs and the group of all miRNAs from the miRBase database statistically overrepresented 8-letter motifs were identified (p-value < 0.01 with Bonferroni correction for multiple comparisons), for these motifs the patterns of the localization in functionally important regions for different types of miRNAs were found. Also, for the group mitomiRs/non-mitomiRs we found the statistically significant features of the miRNA nucleotide context near the Dicer and Drosha cleavage sites (Pearson’s χ2 test of independence for the first three positions of the miRNA, p-value < 0.05). The observed nucleotide frequencies may indicate a more homogeneous pri-miRNA cleavage by the Drosha complex during the formation of the 5’ end of mitomiRs. The obtained results can help to determine the role of the nucleotide signals in the origin, processing, and functions of the mitomiRs.

## Abstract (ru)

МикроРНК – это малые некодирующие РНК, которые регулируют экспрессию генов на посттранскрипционном уровне в цитоплазме, и, таким образом, играют важную роль в большом числе биологических процессов. Последние исследования обнаружили присутствие последовательностей микроРНК не только в цитоплазме, но и внутри митохондрий. Такие микроРНК (так называемые митомиры, mitomiRs) могут иметь ядерное или митохондриальное происхождение, при этом для некоторых из них установлена роль в регулировании функций митохондриальных генов, а для большинства она пока неизвестна. Выявление нуклеотидных сигналов, уникальных для митомиров, может помочь определить эту роль. В нашей работе составлена выборка экспериментально обнаруженных митомиров человека, мыши и крысы. С целью выделения сигналов, которые могут быть ответственны за функционирование митомиров и за их транспортировку в митохондрии или из них, осуществлен контекстный анализ для полученных последовательностей митомиров. Для трех видов в группе данных митомиры/не-митомиры и в группе всех микроРНК из базы miRBase выявлены статистически перепредставленные 8-буквенные мотивы (уровень значимости p <0.01 с учетом поправки Бонферрони на множественность сравнения). Для этих мотивов обнаружены закономерности их локализации в функционально значимых участках для разных типов микроРНК. Для рассматриваемой группы митомиры/не-митомиры также обнаружены статистически значимые особенности нуклеотидного состава последовательностей микроРНК возле границ разрезания комплексами Drosha/ Dicer (критерий независимости χ2 Пирсона для первых трех позиций микроРНК с уровнем значимости p < 0.05). Наблюдаемые частоты нуклеотидов, предположительно, могут указывать на наличие у митоми- ров (в сравнении с не-митомирами) более однородного разрезания прай-миРНК комплексом Drosha при формировании 5’-конца последовательностей. Результаты работы могут быть полезными для выявления сигналов, принимающих участие в возникновении, процессинге и функциях митомиров.

## Introduction

The main pathways of miRNA biogenesis, starting at a cell’s nucleus and ending in the cytoplasm, have been studied quite well to date (Bartel, 2018). Studying the nucleotide context of microRNAs and their precursors (pri-/pre-miRNAs) established the presence of signals that can affect the functions of miRNAs as well as their maturation at different stages of biogenesis. The nucleotide sequence of a miRNA can both directly determine its functions and affect the 5′-end cleavage accuracy by Drosha/Dicer complexes, thus forming sitespecifically modified miRNAs having a shift in the so-called “seed region”, a region from 2 to 7 miRNA nucleotides responsible for its addressing (Starega-Roslan et al., 2015a, b; Rolle et al., 2016).

The presence of motifs in the single-stranded ends (UG; CNNC) and in the basal stem of the pri-miRNAs (CUC/ GHG) or in the terminal loop (GU) of the pre-miRNA hairpin can lead to blocking or, conversely, to facilitating miRNA processing (Auyeung et al., 2013; Fang, Bartel, 2015; Nguyen et al., 2015; Starega-Roslan et al., 2015a, b; Rolle et al., 2016; Vorozheykin, Titov, 2020). Apart from the nucleus and cytoplasm, these small RNA sequences, as well as the proteins of their processing complexes, are found in organelles, for example, in mitochondria (Kren et al., 2009; Bandiera et al., 2011; Wang et al., 2015). These observations show there are possibly new pathways for miRNA biogenesis inside a mitochondrion as well as ways for transportation of mature miRNAs between the cytoplasm and mitochondria by yet unknown transport complexes. The existence of such mitochondrial miRNAs (so-called mitomiRs) raises questions about their evolutionary origin and their functions inside and outside organelles and whether they have the structural features enabling their functions and transportation inside or outside mitochondria.

This paper is a review of published materials devoted to experimentally observed miRNAs in a mitochondria. For selected mitomiRs, their sequences’ contextual features have been evaluated to investigate the possible influence of nucleotide signals on the origin, processing, and functions of the mitomiRs.

## Materials and methods

For our review, miRNA sequences of Homo sapiens, Mus musculus, Rattus norvegicus from the miRBase database (http://miRBase.org, edition 22.1) (Kozomara et al., 2019) were selected. The total number of the included sequences comprised 5398.

The information about mitomiRs was obtained from the articles, whose authors experimentally investigated, applying the RT-qPCR, microarray, qRT-PCR methods, microRNA localization inside and outside the mitochondria of different organisms and tissues (Kren et al., 2009; Bian et al., 2010; Bandiera et al., 2011; Barrey et al., 2011; Mercer et al., 2011; Das et al., 2012; Sripada et al., 2012; Wang et al., 2015). Based on these publications, two sets of sequences were formed for human, mouse, and rat: mitomiRs (652 miRNA sequences observed in mitochondria) and all other miRNAs from the miRBase database (4766 sequences) hereinafter called nonmitomiRs).

To study the features of the sequences of these two groups, a search for statistically overrepresented ( p-value < 0.01 with Bonferroni correction for multiple comparisons) oligonucleotide motifs was carried out using the ARGO software (Vishnevsky, Kolchanov, 2005) to perform a de novo search for motifs in the 15-letter code for mitomiRs/non-mitomiRs sample pairs and for all miRNAs from the miRBase database. When searching for motifs in the microRNAs from the miRBase database, the software estimated the expected proportion of random sequences with a mononucleotide frequency composition similar to that of an analyzed sample containing a motif for random reasons.

For the obtained motifs, an assessment to estimate their similarity within each of the considered groups and between the two groups was performed. For every pair of motifs, the Jaccard similarity coefficient was calculated as Nsimil Ntotal , where Nsimil is the number of all 4-letter nucleotide sequences corresponding to both motifs. The coefficient takes a value from 0 to 1, where 0 indicates a complete difference between the two motifs, and 1 – a complete match. To estimate the probability of obtaining the Jaccard coefficient for random reasons, the method proposed in (Real, Vargas, 1996) was applied where the random value of the Jaccard coefficient is assumed to be distributed according to the binomial law (up to normalization). For the identified motifs (found in 616 mitomiRs and 4043 non-mitomiRs), an analysis of their localization in miRNA sequence and an analysis of the nucleotide context were performed to identify the heterogeneity of miRNA cleavage from a precursor by the Drosha and Dicer complexes. The localization analysis of all microRNAs from the miRBase database involved the random positions selected within the sequences of an analyzed sample and used as a “contrast” sample.

## Results and discussion

In the reviewed publications, 652 unique miRNA identifiers were mentioned. 272 sequences from the found mitomiRs can be characterized as highly reliable, since they were either additionally verified by RT-qPCR/qRT-PCR methods, or in the data of microarray experiments, they were observed in greater numbers inside mitochondria than outside them

It is worth mentioning seven mitomiRs, whose sequences fully present in the human mitochondrial genome: hsa- miR- 1974, hsa-miR-1977, hsa-miR-1978, hsa-miR-4461, hsa- miR- 4463, hsa-miR-4484, hsa-miR-4485-3p, and that can serve as an additional confirmation of their validity. At the same time, due to miRNA sequence and mitochondrial tRNA imposition, references to the following miRNAs such as hsamiR- 1974, hsa-miR-1977, hsa-miR-1978 were removed from the miRBase database. The hsa-miR-4461 microRNA was also removed from the database since the experimental data obtained for it did not meet the miRNA-annotation requirements. Hence, the sequences that did not correspond to the currently known miRNA biogenesis pathways but could be formed through unknown non-canonical pathways had been excluded from the miRBase database.

For further investigation and comparison of mitomiR characteristics, a sample of non-mitomiRs of a total of 4766 sequences was also used in the study. It includes all human, mouse, and rat miRNAs from the considered miRBase database, excluding the selected mitomiRs.

Using the ARGO software (Vishnevsky, Kolchanov, 2005), all the two miRNAs groups (mitomiRs/non-mitomiRs group, and all the miRNAs included in the miRBase database) were analyzed. For each of the groups, 40 (Table 1) and 44 (Table 2) 8-nucleotide IUPAC motifs were selected, each having a statistically significant difference in occurrence in miRNA samples in each of the groups ( p <0.01 with Bonferroni correction for multiple comparisons). For the motifs within each of the groups, as well as for the motifs from different groups, the Jaccard similarity coefficient average value (averaged over motif pairs excluding zero values) for all three calculations did not exceed 0.02.

**Table 1.**
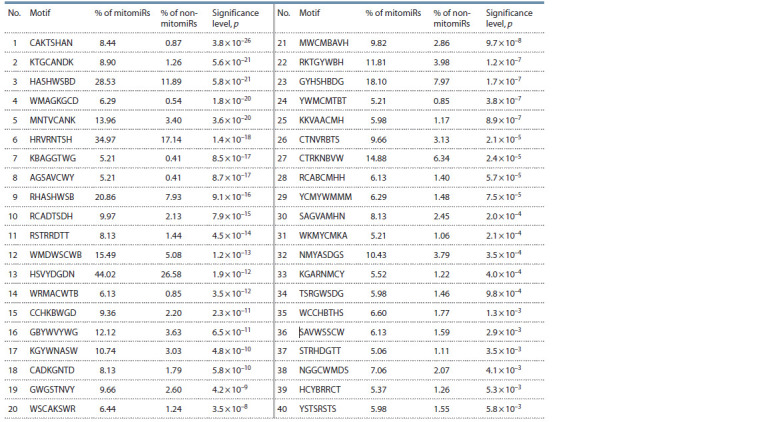
Motifs that have a statistically significant difference in occurrence between the mitomiRs/non-mitomiRs groups Notе. % of mitomiRs is a proportion of mitomiR sequences containing the motif; % of non-mitomiRs is a proportion of non-mitomiR sequences containing the motif. Here and in the Table 2: The table includes only those motifs whose significance level was p < 0.01 with Bonferroni correction for multiple comparisons.

**Table 2.**
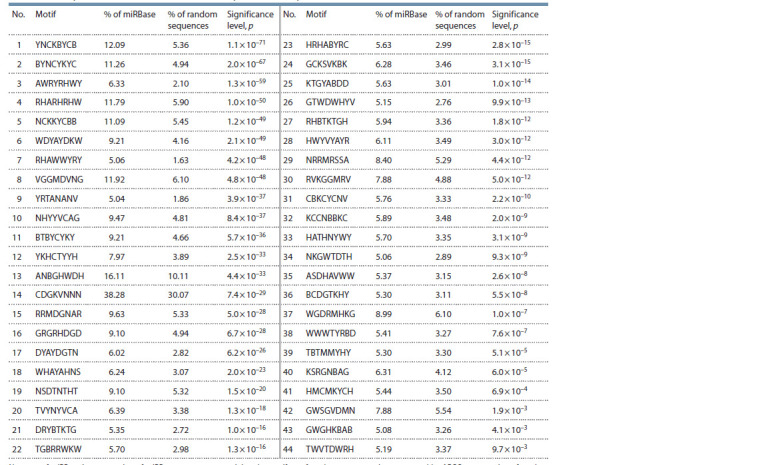
Motifs having a statistically significant difference in occurrence between the group of all miRBase base miRNAs and random sequences of similar mononucleotide composition as analyzed miRNAs Notе. % of miRBase is a proportion of miRBase sequences containing the motif; % of random sequences is an expected by ARGO a proportion of random sequences with a mononucleotide frequency composition similar to analyzed sample containing a motif for random reasons.

For two motifs, KTGCANDK from the mitomiRs/nonmitomiRs group and KTGYABDD from the group of all microRNAs, a maximum Jaccard coefficient of (0.3) and a minimum probability to observe it for random reasons of (0.81) were obtained. These motifs were found in 193 sequences from the mitomiRs/non-mitomiRs group and in 315 sequences in the group of all miRNAs from the miRBase database. Apart these two motifs, the Jaccard coefficient for all other considered pairs did not exceed 0.13 with the probability of observing corresponding coefficients for random reasons being less than 0.001, so the observed data showed a low degree of motif coincidence both within each of the groups and between the two groups.

The observed differences in the nucleotide composition between the samples of mitomiRs and non-mitomiRs can act as specific signals for mitomiR processing, e. g., for recognition and transportation of sequences to/from mitochondria by transport complexes or for the implementation of mitomiR specific functions through direct binding to targets in mitochondrial or cellular DNAs. At the same time, the motifs found in the group of all miRNAs may correspond to the signals common for the processing and functioning of miRNAs, regardless of their localization

**Fig. 1.**
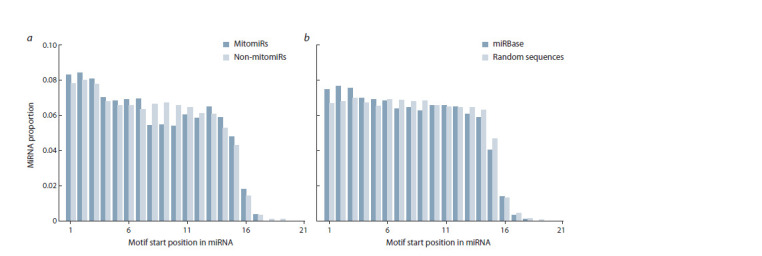
MiRNAs proportion depending on the motif-start positions found in the sequences of mitomiRs and non-mitomiRs (a) and in the miRNAs from the miRBase database (b). For each miRNA with a motif, its position starting from the 5’ end of the sequence was determined. If one motif occurred several times in a microRNA or several motifs occurred once in a microRNA, each occurrence was considered independently and generated a data structure (microRNA, motif position). The graph was normalized for the total number of structures obtained for all motifs. The decrease in observations in the positions whose numbers were greater than 15 was due to the variability in the lengths of miRNA sequences changing from 15 to 28 nucleotides.

For both considered groups the first motif position tended to be located at the beginning of a miRNA (Fig. 1), so the maximum proportion of sequences was observed for the motifs with their start being at positions 1–3. For the obtained observations, a statistically significant dependence of a miRNAsequence type on the positions of a motif start (Pearson’s χ2 test of independence, p-values 4.46 × 10–2 and 6.58 × 10–5 for the mitomiRs/non-mitomiRs group and the miRbase miRNAs/ random positions in miRNAs group, respectively). At the same time, for the mitomiRs, in contrast to the other samples, a significant reduction in the number of miRNAs whose motif start was located at positions 8–10 of a microRNA was observed. A possible reason for that was that the so-called seed region of all miRNAs (both mitomiRs and non-mitomiRs) is the most conservative and significant region in terms of its functionality, and therefore the considered 8-letter conservative motifs often take this region.

In contrast to this, the motifs starting from 8 to 10 position in a miRNA are often localized in the region of the so-called additional seed (~13–16), which is supposedly less conservative in mitomiRs and less often participates in microRNA binding to the target if compared to non-mitomiRs (see Fig. 1, a). However, since the sample of all miRNAs mostly consisted of non-mitomiRs, its observation results approximately coincided with those obtained for non-mitomiRs.

For the detected motifs, different localization patterns within a miRNA sequence were observed. One motif could be observed as in several different miRNAs with different localizations within the sequences (e. g., the KTGCANDK motif with a significance level of p = 5.6 × 10–21 started at position 14 from the 5′ end of the hsa-miR-92a-1-5p mitomiR, and at position 2 from the 5′ end of the mmu-miR-19b-3p mitomiR) as within one miRNA, including cases that did not intersect each other (e. g., in the hsa-miR-33a-5p mitomiR, the KTGCANDK motif occurs twice, starting from position 1 and from position 12 from the 5′ end).

Hence, the variability of motif localization in a miRNAs may indicate both the functional importance of these nucleotide signals for these miRNAs and the possible involvement of the signals in microRNA processing, in particular, in the selection and transportation of mitomiR sequences between a mitochondrion and cytoplasm

**Fig. 2.**
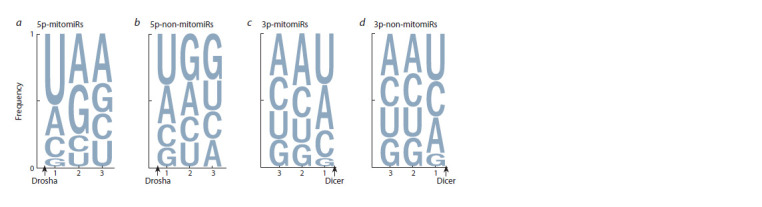
Nucleotide occurrence frequency for the first three positions starting from the 5’ end of the microRNA from 5p- and 3p-branches of pre-miRNA for mitomiRs (a, c) and non-mitomiRs (b, d ) samples in the sequences where motifs were found. The sizes of the letters are proportional to the frequencies. The X-axis displays the position numbers in microRNA, starting from the 5’ end. The arrows show the Dicer or Drosha cleavage sites. For positions 1–3 in a 5p-miRNA and for positions 1–2 in a 3p-miRNA presented a statistically significant dependence of miRNA sequence type on nucleotide occurrence frequency for a considered position (Pearson’s χ2 test of independence, p-value 2.89 × 10–31, 1.03 × 10–28, 1.79 × 10–42, 1.17 × 10–9, 3.23 × 10–10 for respective positions).

For the considered miRNA groups, an increase in the proportion of the sequences where motifs began in positions 1–3 was observed (see Fig. 1), so sequence analysis of this region, but only for those mitomiR and non-mitomiR sequences in which motifs has previously been found, was performed. For the first three positions of the 5′ end of 5p- and 3p-miRNAs, the positional frequencies of nucleotide occurrence were calculated (Fig. 2).

In the mitomiRs from a pre-miRNA’s 5p-branch, U was predominantly observed in the first position and G was very rarely found, while A or G were mainly detected in the second position (see Fig. 2, a). In non-mitomiRs, an increase in the first position of the number of G and A nucleotides and a decrease in the number of U were observed (see Fig. 2, b). For the Drosha cleavage site, an inversion in 2–3 positions between G in non-mitomiRs and A in mitomiRs was detected. For each of the three positions, the nucleotide frequencies showed dependence on a miRNA type (Pearson’s χ2 test of independence, p-values 2.89 × 10–31, 1.03 × 10–28, and 1.79 × 10–42 for the 1, 2 and 3 positions, respectively), while the third position demonstrated the most significant difference in frequencies between miRNA types, in contrast to the first and second ones.

Comparing the observed nucleotide context of the 5′ ends for mitomiRs/non-mitomiRs with the results of a study that investigated pre-miRNA cleavage accuracy by Drosha and Dicer complexes (Starega-Roslan et al., 2015b), it can be assumed that the Drosha cleavage was more accurate for the mitomiRs from the 5p-branch of pre-miRNAs than for nonmitomiRs, in other words, a more homogeneous 5′ end and a corresponding seed region were formed for mitomiRs, which may be the evidence of greater conservatism of mitomiR functions in comparison to those of non-mitomiRs. The detected signals of mitomiR cleavage homogeneity could be an indication either of the possible existence of a more accurate Drosha-like complex for miRNA processing in mitochondria or of the possible compensation of inaccurate cleavage by the nucleotide composition of the pri-miRNA sequences selected for processing by the Drosha complex.

For the 5p-non-mitomiRs, the context shifted towards heterogeneous cleavage, i. e., more active site-specific miRNA modification, and in this case, the non-mitomiRs could act as a functional-variability factor. It can be assumed that mitochondria do not “tolerate” the variability of “their” miRNAs and eliminated regulatory-sequence isoforms in the course of evolution, so the observed mitomiRs may be the remaining conservative sequences that originated in the times mitochondrial ancestors were domesticated.

For mitomiRs and non-mitomiRs from the 3p branch of pre-miRNA, no noticeable differences in the nucleotide context were observed, except for the inversion in the first position of the second and third most popular nucleotides (see Fig. 2, c and d ). Statistically significant dependence of positional nucleotide frequencies on a miRNA type was demonstrated only at the first and second positions (Pearson’s χ2 test of independence, p-values: 1.17 × 1.17 × 10–9 and 3.23 × 10–10, respectively). Comparison of the observed nucleotide frequencies against the results obtained by (Starega-Roslan et al., 2015b) did not allow us to make unambiguous conclusions about the cleavage quality of the 5′ end of the 3p-miRNA by the Dicer complex.

## Conclusion

In the present study, a sample of experimentally confirmed mitomiRs was formed and a nucleotide analysis of their sequences was performed. For the mitomiRs/non-mitomiRs group and the group of all microRNAs from the miRbase database, statistically overrepresented 8-letter IUPAC motifs within miRNA sequences were found. These motifs demonstrated that mitomiR sequences may represent a new, non-canonical class of miRNAs. While the motifs for the mitomiRs/non-mitomiRs group could act as signals for mitomiR processing, e. g., participating in mitomiR transportation to/from mitochondria or for mitomiR function implementation through binding to targets in mitochondrial or cellular DNAs, the motifs of the group of all microRNAs could correspond to the signals common for the processing and functions of miRNAs, regardless of their localization in the

The nucleotide context of the mitomiRs (if compared to that of the non-mitomiRs) near the 5′ end formed by Drosha/ Dicer cleavage could presumably indicate a more uniform formation of the 5′ end of mitomiR sequences and, thus, a more conserved functionality of these sequences.

## Conflict of interest

The authors declare no conflict of interest.
